# Celastrol slows the progression of early diabetic nephropathy in rats via the PI3K/AKT pathway

**DOI:** 10.1186/s12906-020-03050-y

**Published:** 2020-10-23

**Authors:** Yusong Nie, Chengxiao Fu, Huimin Zhang, Min Zhang, Hui Xie, Xiaopei Tong, Yao Li, Zhenyan Hou, Xinrong Fan, Miao Yan

**Affiliations:** 1 Department of Pharmacy, The Second Xiangya Hospital, Central South University, Changsha, 410011 Hunan China; 2 Hunan University of Chinese Medicine, Changsha, 410208 Hunan China; 3 Xianyang Central Hospital, Xianyang, 712000 Shaanxi China; 4 Center of Clinical Pharmacology, The Third Xiangya Hospital, Central South University, Changsha, Hunan 410013 China; 5 First clinical medical college, Shaanxi University of Chinese Medicine, Xianyang, 712000 Shaanxi China; 6 Department of Pharmacy, The Affiliated Yantai Yuhuangding Hospital of Qingdao University, Yantai, 264000 Shandong China; 7 Institute of Basic Theory for Chinese Medicine, China Academy of Chinese Medical Sciences, Beijing, 100700 China

**Keywords:** Celastrol, Diabetic nephropathy, PI3K, AKT, Podocytes, Autophagy, Glomeruli basement membrane

## Abstract

**Background:**

Diabetic nephropathy serves as one of the most regular microvascular complications of diabetes mellitus and is the main factor that causes end-stage renal disease and incident mortality. As the beneficial effect and minute adverse influence of Celastrol on the renal system requires further elucidation, the renoprotective function of Celastrol in early diabetic nephropathy was investigated.

**Methods:**

In high-fat and high-glucose diet/streptozotocin-induced diabetic rats which is the early diabetic nephropathy model, ALT, AST, 24 h urinary protein, blood urea nitrogen, and serum creatinine content were observed. Periodic acid-Schiff staining, enzyme-linked immunosorbent assay, immunohistochemical analysis, reverse transcription-polymerase chain reaction, and western blot analysis were used to explore the renoprotective effect of Celastrol to diabetic nephropathy rats and the underlying mechanism.

**Results:**

High dose of Celastrol (1.5 mg/kg/d) not only improved the kidney function of diabetic nephropathy (DN) rats, and decreased the blood glucose and 24 h urinary albumin, but also increased the expression of LC3II and nephrin, and downregulated the expression of PI3K, p-AKT, and the mRNA level of NF-κB and mTOR.

**Conclusion:**

Celastrol functions as a potential therapeutic substance, acting via the PI3K/AKT pathway to attenuate renal injury, inhibit glomerular basement membrane thickening, and achieve podocyte homeostasis in diabetic nephropathy.

## Background

Diabetic nephropathy (DN), a common microvascular change of diabetes mellitus (DM), is the main cause of end-stage renal disease (ESRD) [1]. Diagnosis of DN is a result of specific pathological, structural, and functional changes observed in the kidneys of DM patients suffering from both type 1 and type 2 diabetes [2]. The clinical phenotype of renal injury is characterized by proteinuria and progressive deteriorations in kidney function. Podocyte injury, glomerular basement membrane (GBM) changes, mesangial cell expansion, endothelial dysfunction, and sterile inflammation are well-known mechanisms that drive kidney disease progression in DN patients [3]. To date, the amelioration of renal injury in DN remains under investigation [4, 5].

Macrophage-regulated inflammation plays a role in progression of DN [6] and suppressing NF-κB signal could ameliorate DN [7, 8]. Notably, transforming growth factor β1 (TGF-β1) could trigger fibrogenesis in chronic kidney disease, including DN [9]. Experimental data showed that PI3K/AKT pathway plays a crucial role in boosting epithelial-mesenchymal transition [10] and blocking autophagy with the downstream signal mTOR [11]. Seriously injured lysosomes and autophagy dysfunction were observed in podocytes of diabetic rats with massive proteinuria [12], indicating autophagy dysfunction is involved in podocyte loss that contributes to proteinuria under diabetic condition. Intriguingly, an adipokine, apelin was reported to inhibited podocyte autophagy and induced podocyte apoptosis through AKT- and mTOR-dependent pathway both in vivo and in vitro, followed by progression of DN [13]. mTOR blockade exerts a beneficial effect in DN, suggesting that the mTOR pathway plays an important pathogenic role in DN [14, 15]. PI3K/AKT, mTOR, and NF-κB are thus possible mediators of DN progression.

**Fig. 1:**
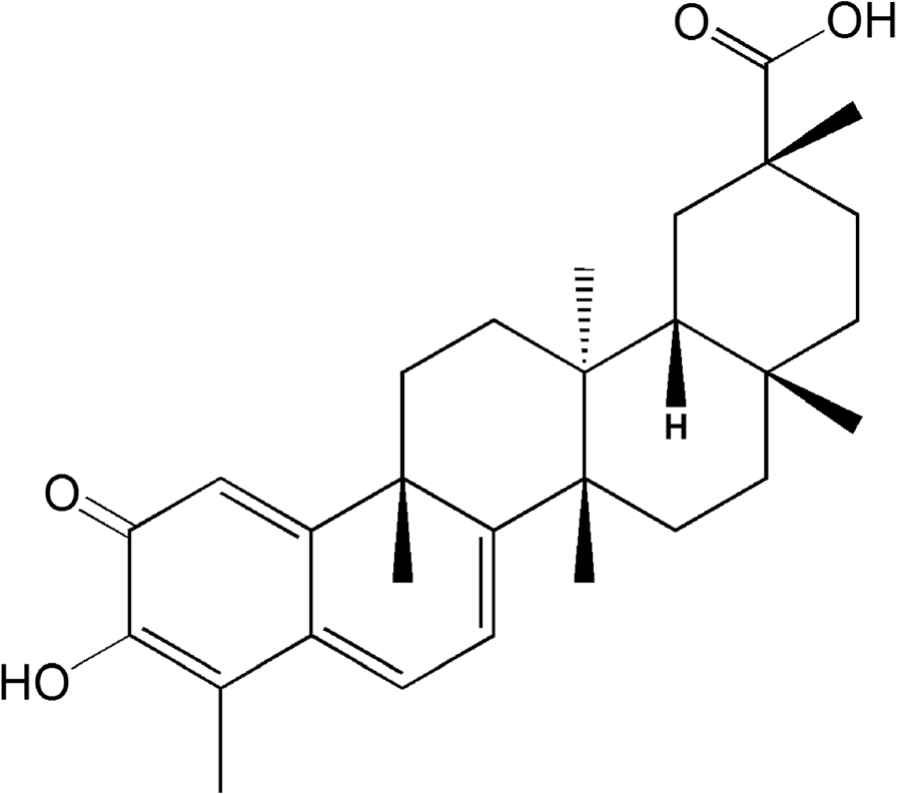
Chemical structure of Celastrol

Celastrol, a pentacyclic triterpenoid (Fig. 1), is a monomeric compound extracted from *Tripterygium wilfordii* Hook F [16]. Recently, Celastrol was demonstrated as a leptin sensitizer and a promising pharmacological substance for the treatment of obesity [17]. Therefore, it continues to receive growing interest. Celastrol may potentiate apoptosis [18], suppress invasion of tumor cells [18], and attenuate angiogenesis to protect against cancer [19]. Celastrol can also prevent mitochondrial ROS in cadmium-administered neuronal cell [20], reduce inflammatory immunity response in obese asthmatic mice, astrocytes and ulcerative colitis-related colorectal cancer [21–23], and inhibit fibrosis in a model of ischemic myocardium and a UUO-induced renal fibrosis BALB/C mice model [24, 25]. Some studies have implied that Celastrol could protect against the occurrence of renal injury in db/db mice and ischemia-reperfusion-induced injury [26, 27]. While according to multiple studies [28–31], Celastrol could protect from LPS-induced inflammation and injury in cells or rats. Contradictorily, Celastrol aggravated lipopolysaccharide (LPS)-induced inflammation and renal injury in mice [32]. Only few systematic reports of the effect of Celastrol on renal amelioration of DN have been presented [33].

In previous study, we found that Celastrol could protect kidney of diabetes type 2 rats by regulating the signal pathway of MAPK/NF-κB, inhibiting inflammation and delaying renal injury [34]. Here, we examined the effect of Celastrol on the progression of renal disease using a rat DN model and investigated the underlying mechanism.

## Methods

### Chemicals and materials

Streptozotocin (STZ, purity > 98.0%, No. 18883–66-4), Celastrol (purity > 99.9%, No. 34157–83-0), and LY294002 (purity > 99.9%, No. 154447–36-6) were purchased from MedChem Express (Shanghai, China). The anesthetic was sodium pentobarbital (Sigma-Aldrich, St. Louis, MO, USA). LC3B (No. 2775 s) and β-actin (No. YM3028) antibodies were obtained from Auragene Cell Signaling Technology Company (Hunan, China). p-AKT (No. AM1009), AKT antibody (No. AM2058) were purchased from ABZOOM (China). Antibody against PI3K (No. ab151549) and GAPDH (No. YM3029) were respectively purchased from Abcam (UK), Immunoway (China). The commercial assay 93 kit (MeiKang, Ningbo, China) used to measure blood urea nitrogen (BUN), serum creatinine (Scr), and urine protein was purchased from the Hunan SJA Laboratory Animal Co., Ltd. (Hunan, China). TGF-β1 ELISA kits were bought from the Nanjing Jiancheng Institute of Biotechnology (Nanjing, China).

### Animal model and experimental intervention

**Fig. 2:**
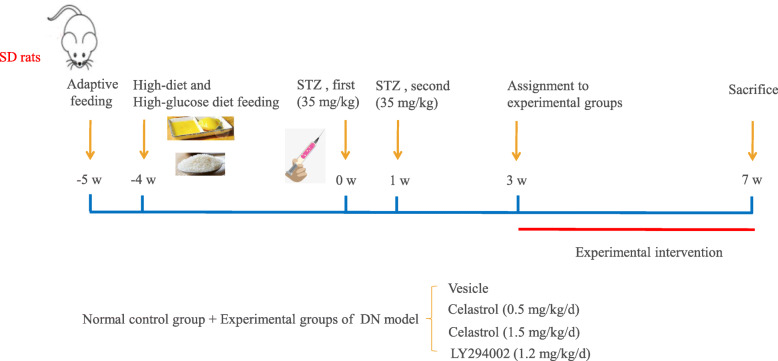
Model adopted to test the experimental design of the study with the DN rat model. Diabetic nephropathy (DN) rats were developed following conventional methods. The rats were subjected to adaptive feeding for the first week and then fed a high-fat and high-glucose diet (sucrose, 15%; cholesterol, 4%; gallate, 0.3%; lard, 10%; yolk powder, 10%; basal food, 60.7%) until the experimental intervention. Rats were randomized and received either vehicle (saline solution) or Celastrol therapy 2 weeks following the second STZ administration, and a consecutive high-fat and high-glucose diet for 4 weeks. Celastrol (0.5 mg/kg/d or 1.5 mg/kg/d) and LY294002 (1.2 mg/kg/d) were orally administered for 4 weeks. The normal control group underwent the same procedures without therapeutic intervention and were fed a standard diet

The male Sprague Dawley (SD) rats (age, 7 weeks old; weight, 180–200 g) used in this study were purchased from the Experimental Animal Center of Hunan Silaike Jingda (Changsha, China), and housed in Specific Pathogen Free (SPF) animal room. The protocol was approved by the ethical committee of Hunan Silaike Jingda (Changsha, China) (authorization number 17/11/176). Every effort was made to relieve the pain and sufferings, and safeguard basic welfare of these animals according to 3Rs principle (Reduction, Replacement and Refinement). Rats were granted free access to drinking water and diet, and were housed at restrained room temperature (23 ± 2 °C), humidity (55% ± 10%) and a 12 h dark/light cycle. To begin (Fig. 2), all rats were subjected to adaptive feeding for 1 week. The rats were then randomized into the normal control (NC) group (*n* = 7) and modeling groups (*n* = 33) based on weight. Rats in the NC group were fed ordinary nutriment while rats in the modeling groups were fed high-fat and high-glucose food (sucrose, 15%; cholesterol, 4%; gallate, 0.3%; lard, 10%; yolk powder, 10%; basal food, 60.7%) for 4 weeks. Then rats in the modeling groups were intraperitoneally injected with streptozotocin (STZ) dissolved in sodium citrate buffer (pH 4.5) twice (a week apart) at a dose of 35 mg/kg, and rats in normal control group were injected the vehicle of STZ, sodium citrate buffer. Three days later, the DM model was determined when fasting glucose (FG) was ≥13.1 mmol/L [35]. If FG met the criteria, the modeled rats were randomly allocated to four groups (*n* = 7) and subjected to corresponding intervention as follows for 4 weeks. The NC group rats were given with saline by gavage as well as the model DN group. Low dose Celastrol (CL, 0.5 mg/kg/d) was administered to the DN + CL group, and high dose (CH, 1.5 mg/kg/d) [17] to the DN + CH group. An intraperitoneal injection with LY294002 (INH, 1.2 mg/kg/d) [36] was administered to the DN + INH group. The vehicle for both Celastrol and LY294002 is saline containing 5% DMSO and 1% Tween-20.

### Animal disposure

After final administration, rats were placed in a metabolic cage and their urine was collected. After 24 h, urine volume was measured and samples were collected for urine albumin examination. Each group of rats was weighed using electronic weighing scales. Following pentobarbital sodium (45 mg/kg, i.p.) anesthesia, limbs were fixed on the operation panel. After performing 75% alcohol disinfection of the abdomen, an abdominal incision was created and a sterile cotton ball with gauze inserted when the abdominal viscera were separated to fully expose the abdominal aorta. Blood was collected from the abdominal aorta using needles and coagulant tubes. After centrifugation at 3500 rpm for 10 min at 4 °C, the upper serum was transferred to a − 20 °C refrigerator for detection. Following cervical dislocation to sacrifice, kidneys were excised, and some of them were frozen in liquid nitrogen and then stored at − 80 °C for further biochemical analysis, while the rest were fixed in 10% (v/v) neutral formalin for histopathologic analysis.

### Physical examination and biochemical parameters detection

We observed the general state of rats before and after model building. We observed mental condition and general changes in daily activities such as water intake, food consumption of each rat in each group throughout the experiment. In each group, the weight of all rats was measured at a fixed time each week using electronic scales. After rats were sacrificed, their kidneys and liver were removed, and the blood and capsule rinsed with PBS buffer and cleaned. Detailed measurements were recorded and the tissues stored in the paraformaldehyde. Kidney index (kidney weight divided by body weight) and liver index (liver weight divided by body weight) were then calculated, and liver function (ALT and AST), kidney function (Scr and BUN), and biochemical indexes detected after 4 weeks of administration. FG was measured with a Sano glucometer. The contents of ALT, AST, GLU, Scr, and BUN were measured using a commercial assay 93 kit (MeiKang, Ningbo, China). Urine volume and urine protein were measured within 24 h following final administration. The right kidney was stored in a frozen storage tube at − 80 °C to prepare for subsequent renal tissue protein determination or western blot (WB) analysis.

### Histopathologic examination

To evaluate morphological changes in renal tissues, the tissues were embedded in paraffin for histological assessments. Samples were subsequently sectioned (4 μm), stained with Periodic acid-Schiff (PAS), and examined under a microscope (Motic, Germany). The PAS-positive areas present in the mesangial region excluding cellular elements were indicated mesangial matrix expansion. The PAS-positive area in the glomerulus was qualitatively analyzed.

### Enzyme linked immunosorbent assay

The concentration of TGF-β1 was assayed using human TGF-β1 Quantikine ELISA Kit. Kidney organ was homogenized (10% w/v) in ice-cold 0.1 M Tris-HCl buffer (pH 7.4). The homogenate was centrifuged at 3000 rpm for 15 min at 4 °C and the resultant supernatant was then collected. The concentration of TGF-β1 measured as instructed by the supplier. Briefly, the standards and test samples were added to the wells of a microplate precoated with a monoclonal antibody specific for TGF-β1, allowing for combination of TGF-β1 and the immobilized antibody at 37 °C for 30 min. After washing away unbound substances, an enzyme-linked monoclonal antibody for TGF-β1 was added to wells and incubated at 37 °C for 30 min. Following washing, substrate solution was added and the absorbance of the presented color was proportional to the amount of TGF-β1. Then absorption was measured at a wavelength of 450 nm by using a Multiskan MS Plate Reader (LabSystems, Inc.; Thermo Fisher Scientific, Inc. USA).

### Immunohistochemical analysis

Immersed in 0.01% sodium citrate buffer (pH 6.0), the renal tissue slices were placed in a microwave for 10 to 15 min, then cooled at 25 °C, and washed with PBS three times for antigen retrieval. The slices were then immersed in 0.1% Triton X-100 for 15 min. To inhibit endogenous peroxidase, all slices were incubated with 3% hydrogen peroxide for 10 min in the dark. These slices were then incubated with 10% goat serum for 60 min at 37 °C, followed by overnight incubation with the primary antibody (anti-Nephrin, 1:200, AM3481, ABZOOM, Auragene) at 4 °C. The negative control sections were incubated with PBS instead of the primary antibody. All sections were incubated with the secondary antibody (sheep anti-rabbit antibody, AuraStain SP rabbit SP kit, P003IH, Auragene) for 60 min at 37 °C and then stained with DAB and hematoxylin. The stained slides were analyzed using a light microscope with brown areas indicating positivity.

### Total RNA extraction and quantitative real-time PCR analysis

Total cellular RNA was extracted using Trizol reagent (Dong sheng, China) according to the provided instructions. Total RNA (4 μg) was reverse transcribed using a PrimeScript RT Reagent Kit (Axygen, USA). Real-time PCR was performed with Power SYBR Green PCR Master Mix (Dong sheng, China) using an Applied Biosystems 7500 Real-Time PCR System. The primer sequences were:

- PI3K, sense-5^′^-CTTGCGAAGTGAGATAGCC-3^′^,
- antisense-5^′^-CTGCGTGAAGTCCTGTAGC-3^′^;
- Akt, sense-5^′^-CACCGCTTCTTTGCCAACAT-3^′^,
- antisense-5^′^-CTGTCATCTTGATCAGGCGGT-3^′^;
- mTOR, sense-5^′^-TCTTTCATTGGAGATGGTTTGGTG-3^′^,
- antisense-5^′^-ATCGGAGGAGATGATTTTCTTTGCC-3^′^;
- NF-κB, sense-5^′^-AGACACAGACGATCGCCACC-3^′^,
- antisense-5^’^AGGTGGTAACACCATGGGGGA − 3^′^;
- β-actin, sense-5^′^-AGGCCCCTCTGAACCCTAAG-3^′^,
- antisense-5^′^- CCAGAGGCATACAGGGACAAC-3^′^.

The relative quantities of each mRNA were determined using the comparative cycle threshold method and normalized to β-actin mRNA.

### Western blot analysis

Renal tissues were homogenized and lysed with RIPA (radioimmunoprecipitation assay) buffer containing protease inhibitors (Auragene, China) on ice. After centrifugation at 13,000×*g* for 20 min at 4 °C, protein concentrations in the samples were determined using the Bradford method. Proteins (20 μg per lane) were loaded onto SDS-polyacrylamide gels and blotted onto methanol-activated PVDF membranes. The membranes were then incubated with PBS containing 0.05% Tween 20 and 5% nonfat dry milk to block nonspecific binding LC3B, PI3K, p-AKT, AKT, β-actin, GAPDH. After blocking in milk for 1 h, the membranes were incubated with the primary antibody against LC3B (1:1000, No. 2775 s, Auragene, China), β-actin (1:1000, Auragene, No. YM3028, Auragene, China), PI3K (1:2000, No. ab151549, abcam, UK), p-AKT (1:1000, No. AM1009, ABZOOM, China), AKT (1:2000, No. AM2058, ABZOOM, China), GAPDH (1:20000, No. YM3029, Immunoway, China) at 4 °C overnight. β-actin and GAPDH were used as an internal control. Incubation with the appropriate secondary antibodies such as sheep anti-rabbit antibody (anti-LC3B, anti-PI3K, anti-p-AKT, anti-AKT) or sheep anti-mouse (anti-β-actin and anti-GAPDH) antibody (1: 15000) was performed for 60 min on a shaking bed. Protein bands were visualized using ECL kit (P020WB, Amersham Biosciences, Auragene, China). Densitometry analyses of the bands were quantified with Image J software.

### Statistical analysis

All values are expressed as mean ± standard error of mean (SEM) of the experiment numbers (*n* = 7). One-way analysis of variance (ANOVA), followed by Turkey’s multiple comparison test was used to analyze statistical differences between different groups, (GraphPad Prism 6.0). *p <* 0.05, statistically significant; *p <* 0.01, very significant; and *p <* 0.001, excessively significant.

## Results

### Effect of Celastrol on body weight, 24 h urinary volume, blood glucose in STZ-induced DN rat fed high fat and high glucose diet

**Fig. 3:**
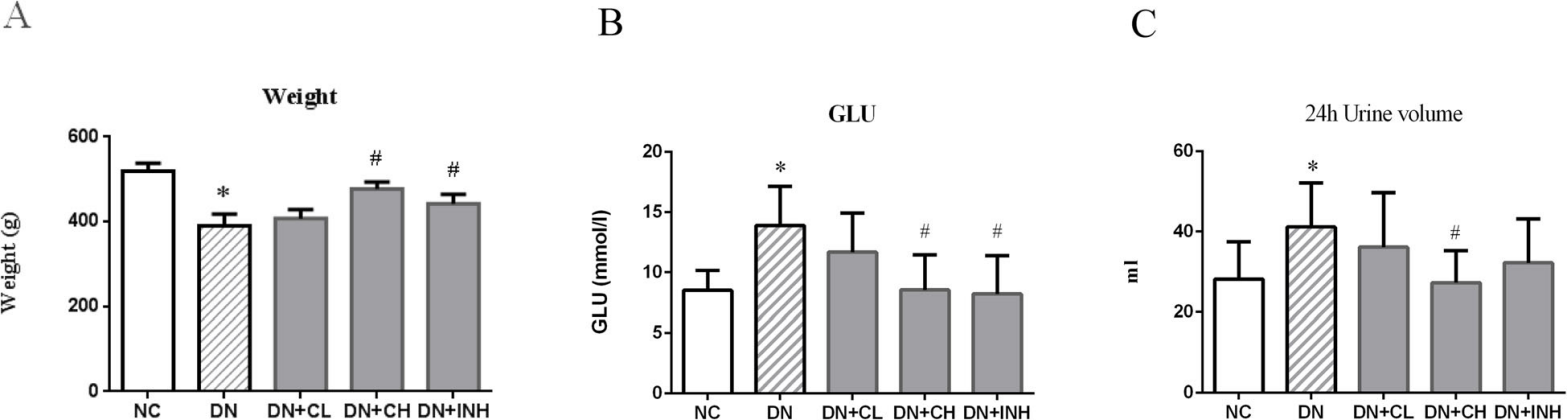
The effects of Celastrol on weight and blood glucose in the DN rat model. **a** body weight and **b** blood glucose, **c** urinary volume. NC group, normal control rats; DN group, high-fat and high-glucose diet-fed STZ-induced DN rats. DN + CL group, DN rats were given by gavage low dose (0.5 mg/kg) Celastrol. DN + CH group, DN rats were given by gavage high dose (1.5 mg/kg/d) Celastrol. DN + INH group, DN rats were intraperitoneally injected with LY294002 (1.2 mg/kg/d). Following the once per day administration of Celastrol or LY294002 for 4 weeks for the intervention, rats were sacrificed. Prior to sacrificing, body weight and blood glucose were determined. Values are presented as mean ± SEM. * *p <* 0.05, ** *p <* 0.01 compared to NC group, ^#^ *p <* 0.05, ^##^ *p <* 0.01 compared to DN group. DN, diabetic nephropathy. We used the one-way analysis of variance (ANOVA), followed by Turkey’s multiple comparison test to analyze the differences

Rats in the DN group exhibited lower body weight and higher 24 h urinary volume and blood glucose when compared to the NC group. These differences were statistically significant (*p <* 0.05). The body weight was significantly increased compared to the DN group (*p <* 0.05), while 24 h urinary volume in DN + CH group and blood glucose in the DN + CH and DN + INH groups were significantly decreased compared to DN group (*p <* 0.05). But body weight in the DN + CL group was virtually unchanged, and the 24 h urinary volume in the DN + CL group and blood glucose in the DN + CL group were slightly decreased with no significant difference compared to the DN group (Fig. 3a-c). These results demonstrated that Celastrol treatment could ameliorate weight, 24 h urinary volume and glucose in rats with early DN.

### Effect of Celastrol on liver index and liver biochemical parameters in DN rat model

**Fig. 4:**
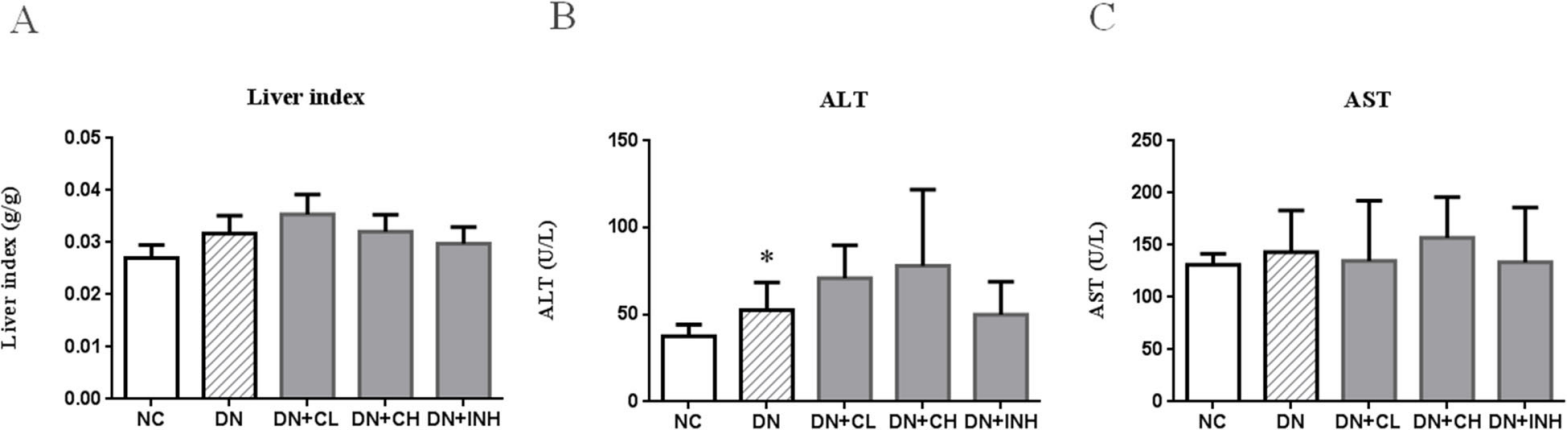
The effects of Celastrol on liver function and liver index in the DN rat model. **a** liver index, **b** ALT, and **c** AST. NC group, normal control rats; DN group, high-fat and high-glucose diet-fed STZ-induced DN rats. DN + CL group, DN rats were given by gavage low dose (0.5 mg/kg/d) Celastrol; DN + CH group, DN rats were given by gavage high dose (1.5 mg/kg/d) Celastrol. DN + INH group, DN rats were intraperitoneally injected with LY294002 (1.2 mg/kg/d). Following the once per day administration of Celastrol or LY294002 for 4 weeks for the intervention, rats were sacrificed and the liver index assayed. Prior to sacrificing, ALT and AST were determined. Values are presented as mean ± SEM. * *p <* 0.05, ** *p <* 0.01 compared to NC group, ^#^ *p <* 0.05, ^##^ *p <* 0.01 compared to DN group. DN, diabetic nephropathy. We used the one-way analysis of variance (ANOVA), followed by Turkey’s multiple comparison test to analyze the differences

ALT and AST levels were assessed as markers of liver function. In contrast to the NC group, liver index and AST levels did not significantly change in the DN group. An exception was the ALT level (*p <* 0.05). With drug intervention, however, the liver index, ALT, and AST levels in the DN + CL, DN + CH, and DN + INH groups were close to those observed in the DN group (Fig. 4a-c).

### Effect of Celastrol on renal index and renal biochemical parameters

**Fig. 5:**
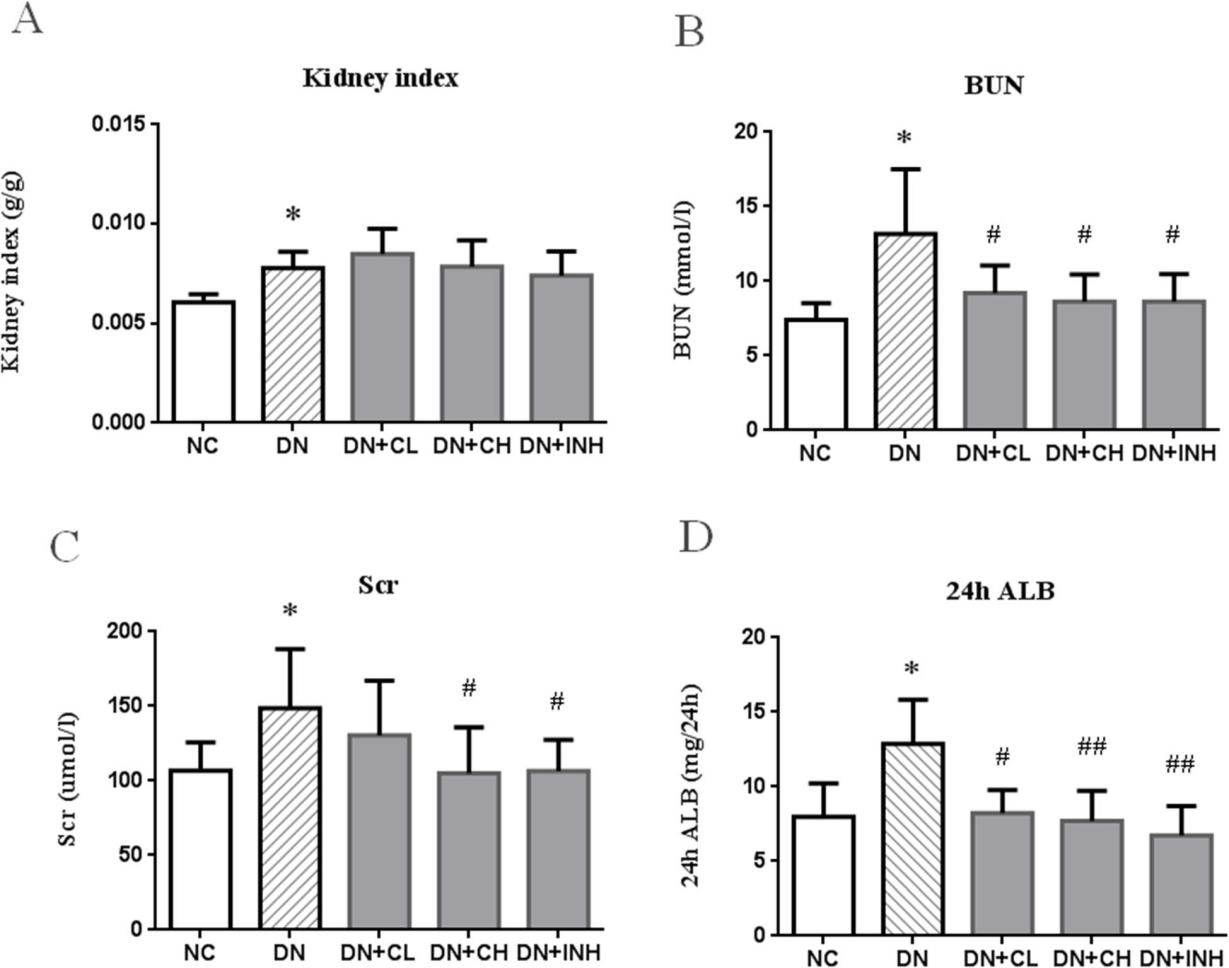
The effects of Celastrol on renal function and kidney index in the DN rat model. **a** kidney index, **b** BUN, **c** Scr, and **d** 24 h ALB. NC group, normal control rats; DN group, high-fat and high-glucose diet-fed STZ-induced DN rats. DN + CL group, DN rats gavaged with low dose (0.5 mg/kg) Celastrol. DN + CH group, DN rats were given by gavage high dose (1.5 mg/kg/d) Celastrol. DN + INH group, DN rats intraperitoneally were injected with LY294002 (1.2 mg/kg/d). Following the once per day administration of Celastrol or LY294002 for 4 weeks for the intervention, rats were sacrificed and the kidney index assayed. Prior to sacrificing, BUN, Scr, and 24 h urinary ALB were determined. Values are presented as mean ± SEM. * *p <* 0.05, ** *p <* 0.01 compared to NC group, ^#^ *p <* 0.05, ^##^ *p <* 0.01 compared to DN group. DN, diabetic nephropathy. We used the one-way analysis of variance (ANOVA), followed by Turkey’s multiple comparison test to analyze the differences

Kidney index in the DN group was significantly increased when compared to that in the NC group (*p* < 0.05). However, kidney index for rats in the DN + CL, DN + CH, and DN + INH groups was very close to that found in the DN group (Fig. 5a). BUN, Scr, and 24 h urinary ALB were denoted as markers of renal function. These markers were significantly elevated in rats in the DN group compared to rats in the NC group (*P* < 0.05). Scr in the DN + CL group showed little change. With Celastrol or LY294002 treatment, BUN, Scr, and 24 h urinary ALB levels were significantly lowered in the modelling groups when compared to the DN group (*p* < 0.05 or < 0.01) (Fig. 5b-d).

### Celastrol relieves renal histopathological injury

**Fig. 6:**
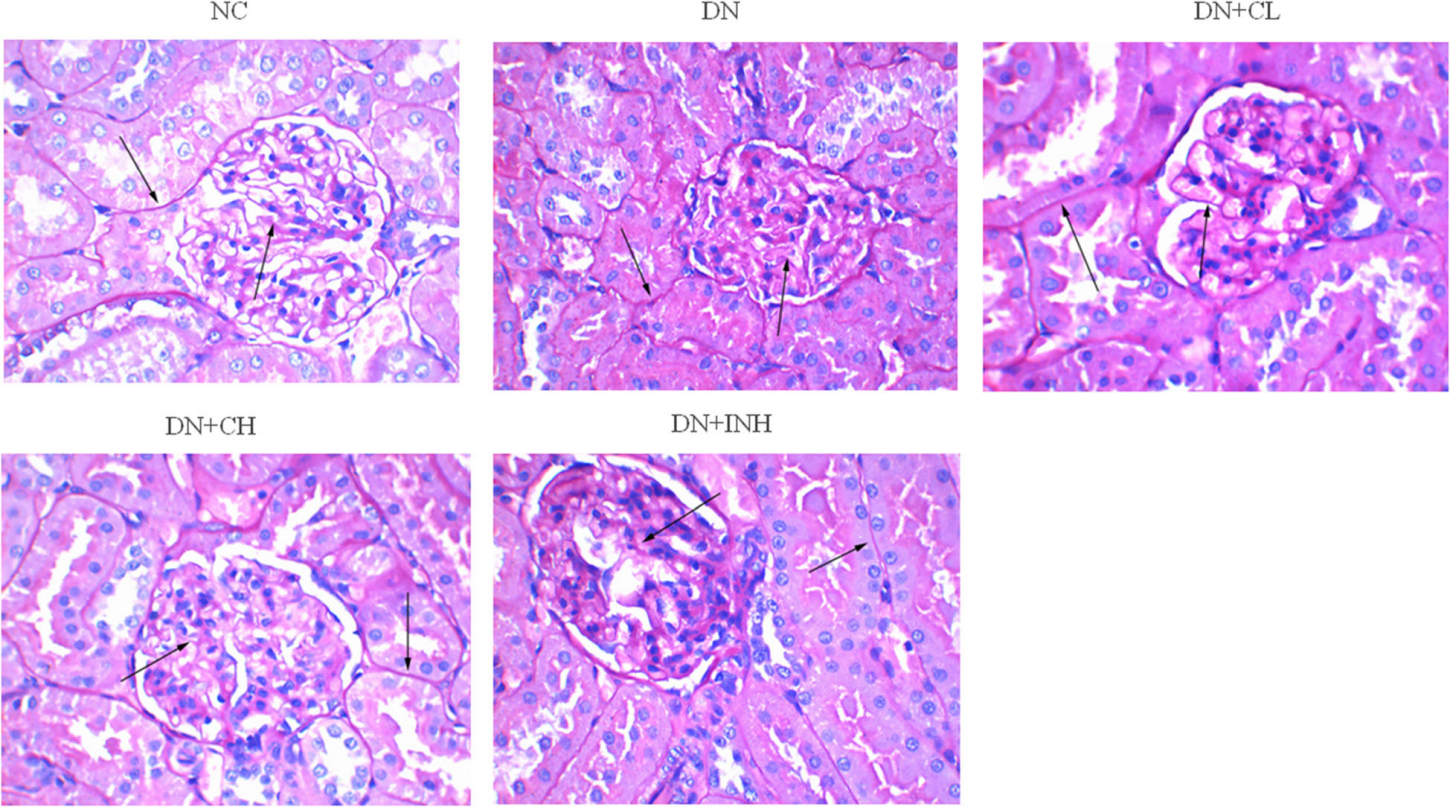
The effects of Celastrol on the renal histopathological status in the DN rat model using PAS staining. Original magnification is × 400. NC group, normal control rats; DN group, high-fat and high-glucose diet-fed STZ-induced DN rats. DN + CL group, DN rats were given by gavage low dose (0.5 mg/kg) Celastrol. DN + CH group, DN rats were given by gavage high dose (1.5 mg/kg/d) Celastrol. DN + INH group, DN rats intraperitoneally were injected with LY294002 (1.2 mg/kg/d). Following the once per day administration of Celastrol or LY294002 for 4 weeks for the intervention, rats were sacrificed and the renal histopathological status assayed by PAS staining. Values are presented as mean ± SEM. * *p <* 0.05, ** *p <* 0.01 compared to NC group, ^#^ *p <* 0.05, ^##^ *p <* 0.01 compared to DN group. DN, diabetic nephropathy. We used the one-way analysis of variance (ANOVA), followed by Turkey’s multiple comparison test to analyze the differences

Renal histopathological results (Fig. 6) were observed using PAS staining of the kidney tissue. Compared to the NC group which showed normal glomerular structure, the DN group showed a lighter blue stain, indicating collagen deposition, thickened GBM, mesangial matrix expansion, decreased visibility of capillary lumen, tubular atrophy, interstitial fibrosis, and extensive glomerular. The DN + CL, DN + CH, and DN + INH groups exhibited mild blue staining, indicating visible capillary lumen and reversed occlusion of the capillary network. Of the modelling groups, the DN + CH group experienced the best restoring effect. The black arrow denotes the glycogen.

### Celastrol inactivates the PI3K/AKT signaling pathway

**Fig. 7:**
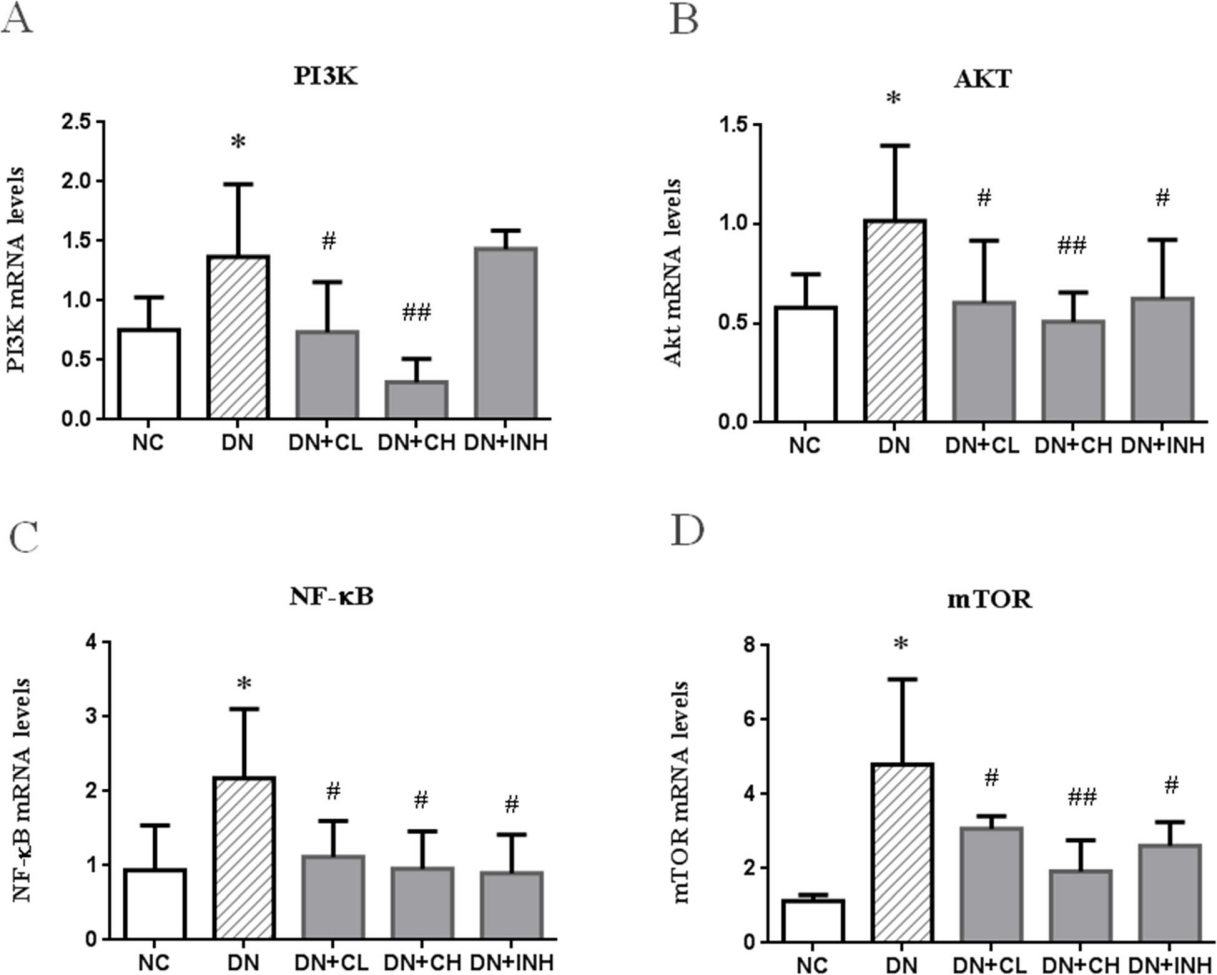
The effects of Celastrol on PI3K, AKT, NF-κB, and mTOR in the DN rat model using RT-PCR. **a** PI3K, **b** AKT, **c** NF-κB, and **d** mTOR. NC group, normal control rats; DN group, high-fat and high-glucose diet-fed STZ-induced DN rats. DN + CL group, DN rats were given by gavage low dose (0.5 mg/kg) Celastrol. DN + CH group, DN rats were given by gavage high dose (1.5 mg/kg/d) Celastrol. DN + INH group, DN rats were intraperitoneally injected with LY294002 (1.2 mg/kg/d). Following the once per day administration of Celastrol or LY294002 for 4 weeks for the intervention, rats were sacrificed and the renal tissues evaluated. The mRNA levels of PI3K and AKT were determined using RT-PCR. Values are presented as mean ± SEM. * *p <* 0.05, ** *p <* 0.01 compared to NC group, ^#^ *p <* 0.05, ^##^ *p <* 0.01 compared to DN group. DN, diabetic nephropathy. We used the one-way analysis of variance (ANOVA), followed by Turkey’s multiple comparison test to analyze the differences

**Fig. 8:**
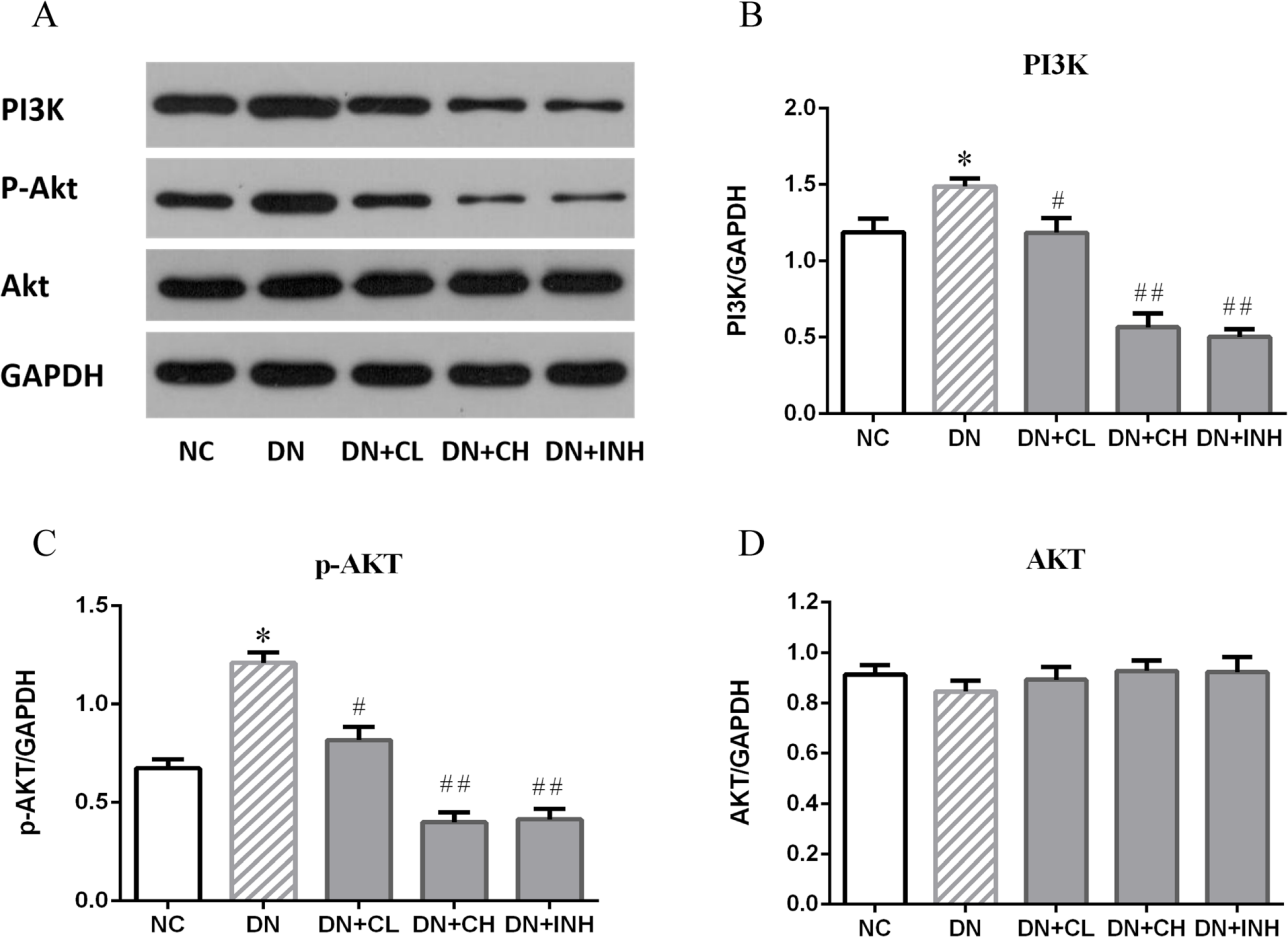
The effects of Celastrol on PI3K (**b**), p-AKT (**c**), AKT (**d**) protein in the DN rat model based on a WB assay. **a** Western blot images of PI3K/AKT pathway. DN group, high-fat and high-glucose diet-fed STZ-induced DN rats. DN + CL group, DN rats were given by gavage low dose (0.5 mg/kg) Celastrol. DN + CH group, DN rats were given by gavage high dose (1.5 mg/kg/d) Celastrol. DN + INH group, DN rats were intraperitoneally injected with LY294002 (1.2 mg/kg/d). Following the once per day administration of Celastrol or LY294002 for 4 weeks for the intervention, rats were sacrificed and the PI3K, p-AKT, AKT protein levels determined by a WB assay of renal tissues. The results were quantified by normalizing to GAPDH. * *p <* 0.05, ** *p <* 0.01 compared to NC, ^#^ *p <* 0.05, ^##^ *p <* 0.01 compared to DN. DN, diabetic nephropathy. We used the one-way analysis of variance (ANOVA), followed by Turkey’s multiple comparison test to analyze the differences

Based on the RT-PCR and western blot results (Figs. 7a, b, 8a-d), upregulation of PI3K and AKT is proposed to play a pivotal role in diabetic renal injury as both the mRNA level of PI3K/AKT and the protein expression of PI3K/p-AKT in the DN group were significantly increased when compared to the NC group (*p <* 0.05). The DN + CL and DN + CH experienced significant attenuation of PI3K/AKT upregulation in contrast to the DN group (*p <* 0.05). It is noteworthy that the DN + CH group exhibited the maximum PI3K/AKT inactivation effect (*p <* 0.01).

### Celastrol reduces the mRNA levels of NF-κB and mTOR

Based on the RT-PCR results (Fig. 7c, d), upregulation of NF-κB and mTOR is proposed to play a crucial role in diabetic renal injury. This is because a significant increase in the mRNA levels of NF-ΚB and mTOR was observed in the DN group when compared to the NC group (*p <* 0.05). After drug intervention, the upregulation of NF-κB and mTOR was significantly attenuated as observed in the DN + CL, DN + CH, and DN + INH groups when compared to the DN group (*p <* 0.05 or < 0.01).

### Celastrol inhibits TGF-β1 protein level

**Fig. 9:**
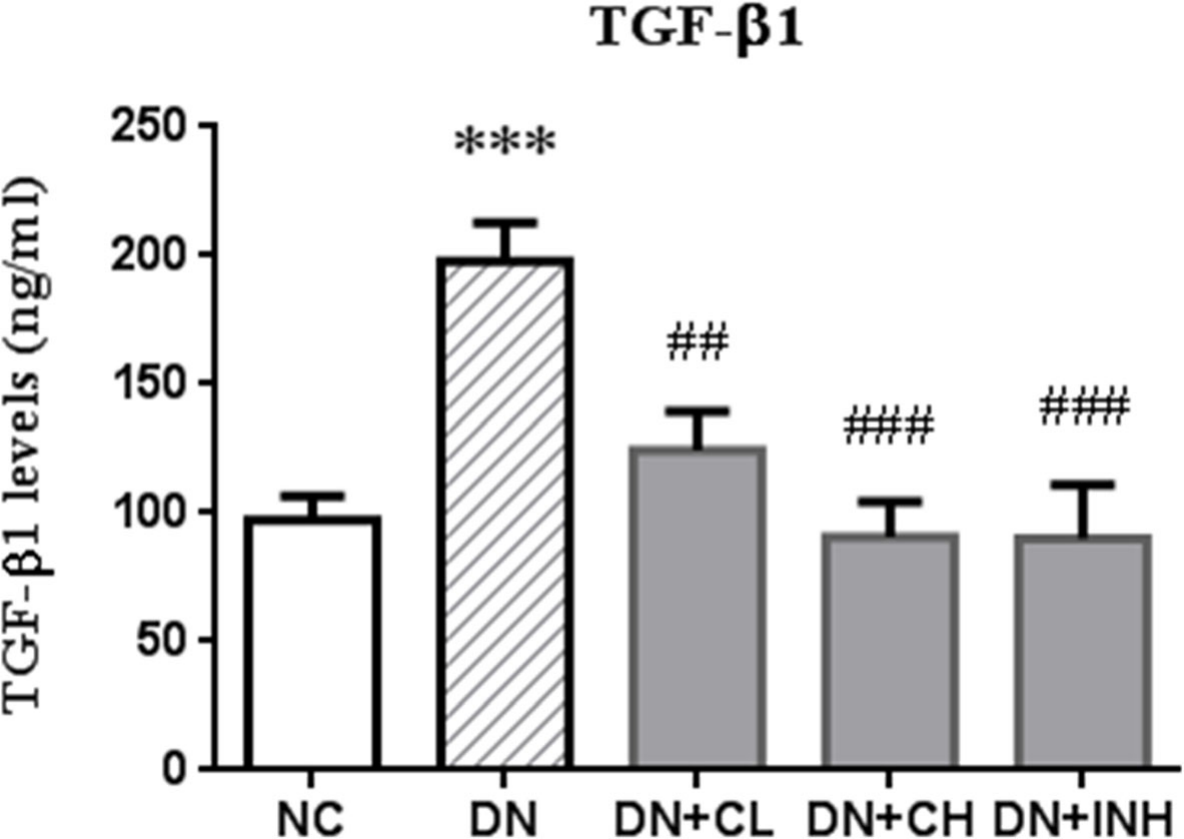
The effects of Celastrol on TGF-β1 levels in the DN rat model measured by ELISA. NC group, normal control rats; DN group, high-fat and high-glucose diet-fed STZ-induced DN rats. DN + CL group, DN rats were given by gavage low dose (0.5 mg/kg) Celastrol. DN + CH group, DN rats were given by gavage high dose (1.5 mg/kg/d) Celastrol. DN + INH group, DN rats were intraperitoneally injected with LY294002 (1.2 mg/kg/d). Following the once per day administration of Celastrol or LY294002 for 4 weeks for the intervention, rats were sacrificed and the TGF-β1 protein levels determined using an ELISA of the renal tissues. *** *p <* 0.001 compared to NC, ^##^ *p <* 0.01, ^###^ *p <* 0.001 compared to DN. DN, diabetic nephropathy. We used the one-way analysis of variance (ANOVA), followed by Turkey’s multiple comparison test to analyze the differences

The ELISA results (Fig. 9) indicated that the protein level of TGF-β1, the pro-fibrosis factor, was significantly upregulated in the renal tissue of rats in the DN group compared to the NC group (*p* < 0.01). Moreover, the level of TGF-β1 was also shown to significantly decrease in the DN + CL, DN + CH, and DN + INH groups compared to the DN group (*p* < 0.01). This finding implies that Celastrol may function as a blocker in the progression of renal injury via TGF-β1 downregulation in DN rats.

### Celastrol treatment increases nephrin expression

**Fig. 10:**
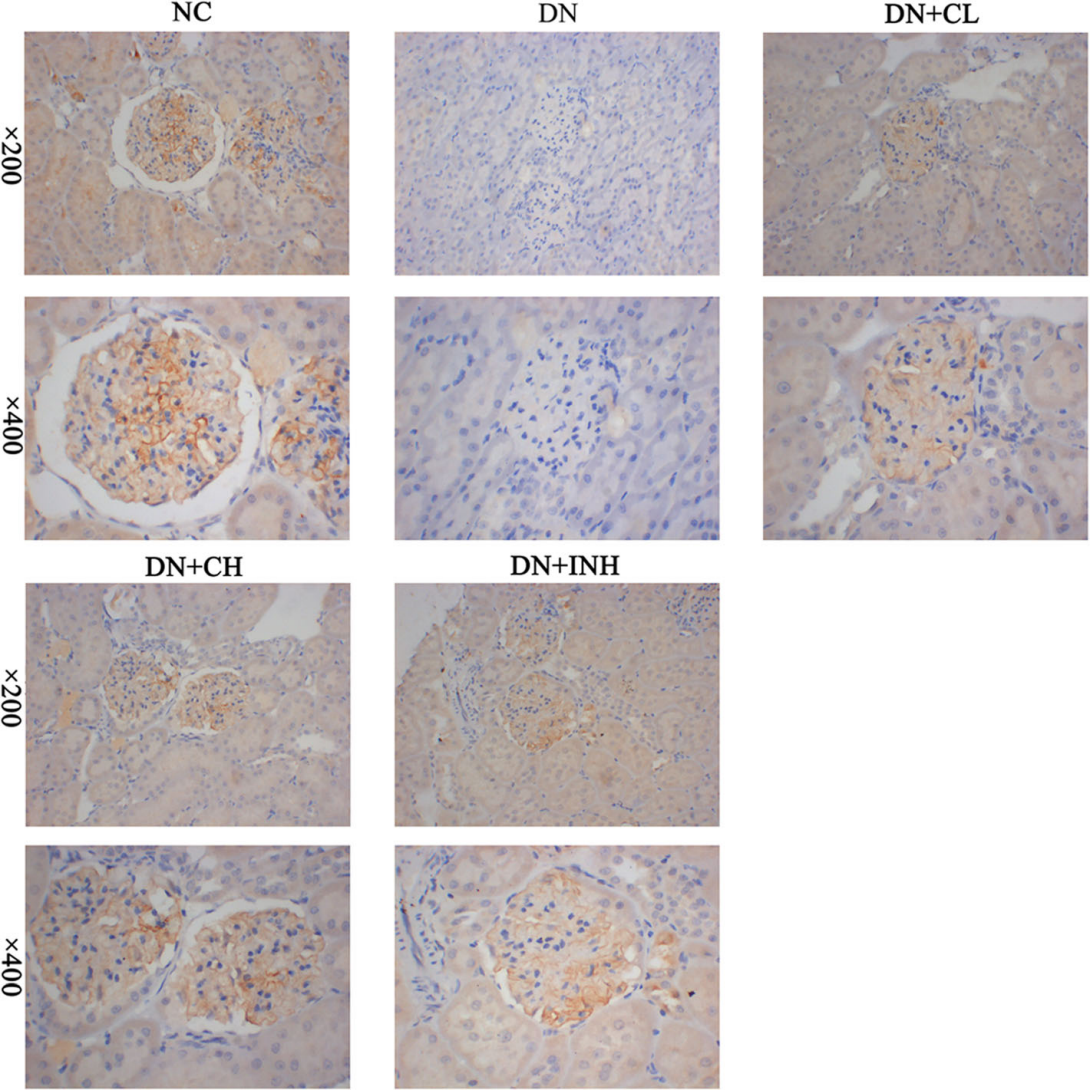
The effects of Celastrol on nephrin protein levels in the DN rat model using the IHC method. The original magnification is × 200 and × 400. NC group, normal control rats; DN group, high-fat and high-glucose diet-fed STZ-induced DN rats. DN + CL group, DN rats were given by gavage low dose (0.5 mg/kg) Celastrol. DN + CH group, DN rats were given by gavage high dose (1.5 mg/kg/d) Celastrol. DN + INH group, DN rats were intraperitoneally injected with LY294002 (1.2 mg/kg/d). Following the once per day administration of Celastrol or LY294002 for 4 weeks for the intervention, rats were sacrificed nephrin protein levels assayed by IHC method of the renal tissues. DN, diabetic nephropathy. We used the one-way analysis of variance (ANOVA), followed by Turkey’s multiple comparison test to analyze the differences

IHC results (Fig. 10) indicated that signals for nephrin were mainly localized in the glomeruli. The intensity and area of nephrin staining were significantly decreased in the glomeruli of rats in the DN group compared to the NC group. Celastrol and LY294002 treatment, however, significantly reversed these consequences as observed in the DN + CL, DN + CH, and DN + INH groups. Compared to the NC group, increased nephrin expression, which appeared as a darker brown color, was evident in the DN + CH and DN + INH groups.

### Celastrol promotes autophagy

**Fig. 11:**
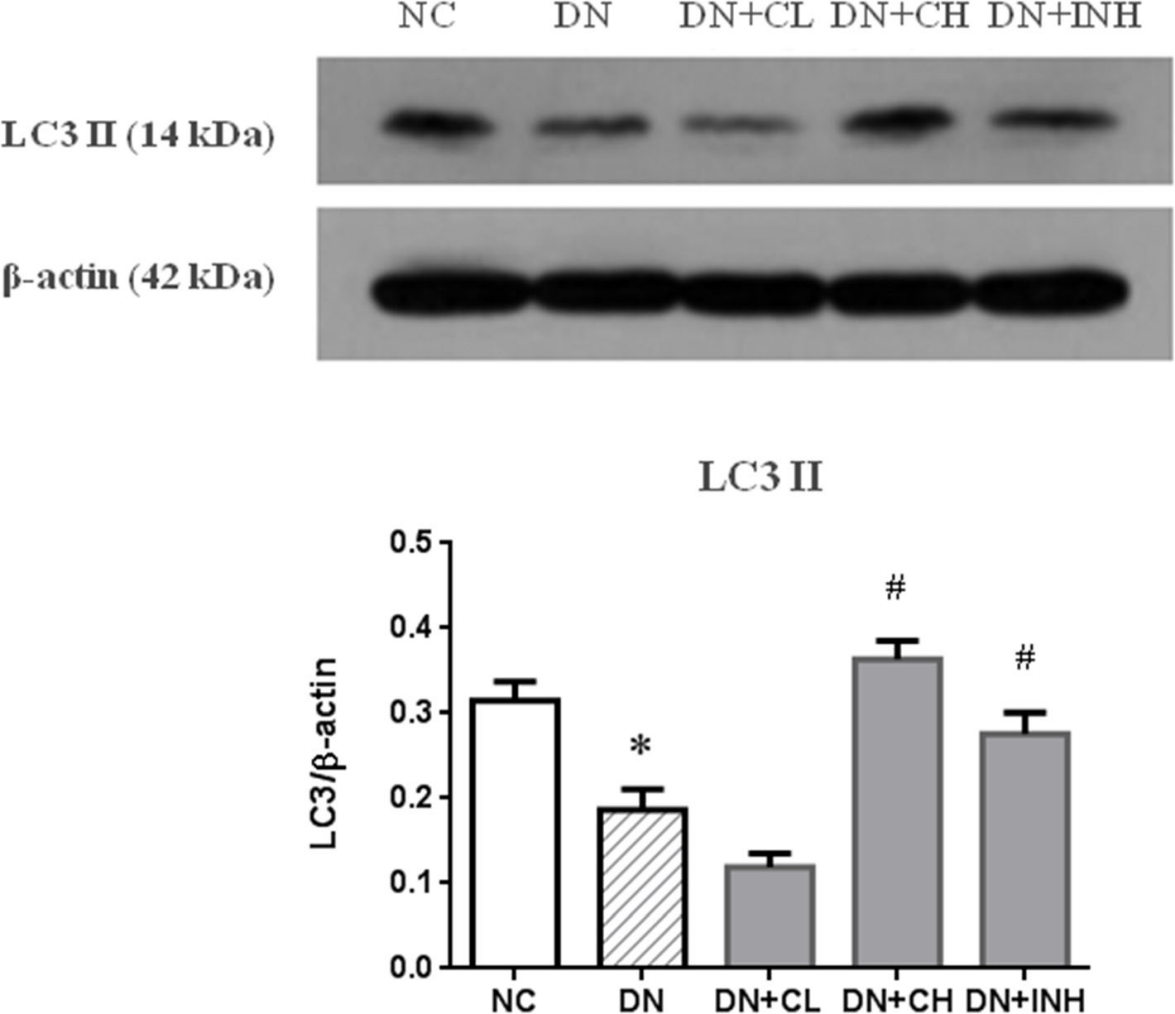
The effects of Celastrol on LC3II (B) protein in the DN rat model based on a WB assay. (A) Western blot images of LC3II. DN group, high-fat and high-glucose diet-fed STZ-induced DN rats. DN + CL group, DN rats were given by gavage low dose (0.5 mg/kg) Celastrol. DN + CH group, DN rats were given by gavage high dose (1.5 mg/kg/d) Celastrol. DN + INH group, DN rats were intraperitoneally injected with LY294002 (1.2 mg/kg/d). Following the once per day administration of Celastrol or LY294002 for 4 weeks for the intervention, rats were sacrificed and the LC3II protein levels determined by a WB assay of renal tissues. The results were quantified by normalizing to β-actin. * *p <* 0.05, ** *p <* 0.01 compared to NC, ^#^ *p <* 0.05, ^##^ *p <* 0.01 compared to DN. DN, diabetic nephropathy. We used the one-way analysis of variance (ANOVA), followed by Turkey’s multiple comparison test to analyze the differences

WB analysis (Fig. 11a, b) showed that the expression of LC3II, the autophagy-related marker, was downregulated in the renal tissue of rats in the DN group when compared to the NC group (*p* < 0.01). LC3II expression in the DN + CL group did not exhibit any evident alteration when compared to the DN group. Nevertheless, treatment with high dose Celastrol or LY294002 may improve LC3II protein level and Celastrol may act as an effective pro-autophagy agent by increasing the expression of pro-autophagy proteins in DN rats.

## Discussion

As a primary feature of DN, persistent proteinuria serves as an independent risk factor for the progression and relative cardiovascular events. Therefore, improving proteinuria is as important as blood glucose control and blood pressure reduction in DN patients. *Tripterygium wilfordii* Hook F extract may function as a potentially effective and safe agent to treat type 2 DM-induced nephropathy, especially for the reduction of urine protein level [33, 37, 38]. As an active ingredient, Celastrol has been focused on by increasing studies including its renal protection function [39].

With the increasing understanding of the molecular pathophysiology of human DN, although targeting additional genes for knockout in isolation or combination may refine animal models, it has been recognized that complete abrogation of gene expression is rarely observed in human diseases [40]. Therefore, the use of animal models is limiting in the ability to grasp DN pathogenesis. This is partly because a model exhibiting all important traits of human diseases does not exist [41, 42]. According to studies reported before [43, 44], the selected SD rats were fed a high-fat and high-glucose diet for 4 weeks. Rats were intraperitoneally injected with STZ (35 mg/kg/d) twice a week. A week later, all modeled rats were administered Celastrol and LY294002 for 4 weeks. The model was evaluated as type 2 DN by referring to previous reports [45, 46]. The type 2 diabetic rats in our study could be determined to have early DN due to the important characteristic of a significant increase in urinary albumin excretion.

In the present study, pretreatment with Celastrol did not produce evident changes in serum ALT and AST activities, and liver index. Thus, we concluded that the implemented dose was safe despite the reported hepatotoxicity [32]. Renal features such as BUN, Scr, and 24 h ALB were, however, increased in the DN group and intervention with Celastrol and LY294002 significantly reversed these increases. Celastrol administration also ameliorated the histopathological changes. Celastrol could therefore prevent the progression of early DN as shown using the rat model.

PI3K/AKT was found to be highly expressed [47], and is the key signal protein mediating many biological effects in DN [48]. TGF-β1, a profibrosis cytokine accompanied by fibronectin and collagen is one of the pathological hallmarks of renal fibrosis in diabetic nephropathy [49, 50]. It was reported that PI3K/AKT pathway may play a critical role in epithelial-mesenchymal transition, which is an important factor inducing renal injury in DN [51]. Moreover, podocyte injury is caused by exosomes derived from high glucose-induced glomerular mesangial cells via TGF-β1-PI3K/AKT pathway [52]. Tubulointerstitial fibrosis was reduced via blockade of PI3K/AKT-induced TGF-β1 expression in db/db mice [53]. Consistently, increased TGF-β1 in DN was reversed by Celastrol and LY294002. It has been clarified that NF-κB is primarily activated by hyperglycemia, oxidative stress, and inflammatory cytokines, thereby leading to diabetic complications, including DN, with other stress-sensitive pathway [54]. Celastrol inhibited NF-κB mRNA level in DN, which is at least a sign that Celastrol could protect against DN through NF-κB-regulating inflammation [34]. In addition, mTOR plays an negative role in mediating autophagy by phosphorylating multiple autophagy proteins, resulting in blockade of the initiating kinase of autophagy, ULK1 (the mammalian orthologs of ATG1, an autophagy-initiating kinase) under high-glucose condition [55]. A study that mTOR activation is crucial for development of podocytes under homeostatic condition, and dysregulated mTOR complex1 (mTORC1) activation causes a series of changes of podocytes in diabetic mice was reported. Progression of glomerulosclerosis in DN and podocyte injury could be hindered by genetically inhibiting the activation of mTOR [15]. In long-term high glucose-administered podocytes, autophagy was inhibited, which was reversed by rapamycin, the inhibitor of mTORC1 [56]. Similarly, rapamycin could also attenuate the renal injury in diabetic mice via restoring autophagy [57]. Hence, blunted autophagy is involved in pathogenesis in DN and restoring autophagy could be a therapeutic strategy for DN [58]. Consistent with the previous report [55, 59], our exploratory research showed that the protein level of LC3II (the autophagy marker) was inhibited and mTOR was enhanced in DN, which was reversed by Celastrol and LY294002. Accordingly, we propose Celastrol probably exhibits renoprotective effect in DN by PI3K/AKT/mTOR/autophagy axis. What’s more, nephrin was the first identified endogenous slit diaphragm molecule that could protect from apoptosis of murine podocytes [60]. Thus, increased nephrin by Celastrol may act as a signal transduction pathway to inhibit podocyte loss. However, under diabetic condition, autophagy activation could be regulated by other nutrient-sensing signal pathways and diabetes-induced intracellular events such as oxidative stress, endoplasmic reticulum stress, hypoxia, accumulation of advanced glycation end-products and so on [61]. So, the role of autophagy in the protective effect of Celastrol on DN remains to be elucidated.

Limitations such as time, energy, and research funding existed in this study. Extrapolating the results on podocyte function may be improved using cell culture systems. In addition, other pathological mechanisms such as endothelial dysfunction should be investigated to elucidate the role of Celastrol in preventing renal injury in early DN. The inadequacies listed herein will be addressed in further research.

## Conclusions

In the present study, we evaluated the protective effects of Celastrol on the progression of renal injury in early-stage DN in rats. By inhibiting PI3K/AKT, Celastrol downregulated the expression of NF-κB which protects against GBM thickening and reduces mTOR expression upstream of autophagy, leading to podocyte homeostasis. Celastrol should be considered as a potential natural resource to develop kidney protective agents that target the incidence of renal disease progression in patients with DN.

## Supplementary information

**Additional file 1.**

## Data Availability

The datasets during and/or analysed during the current study available from the corresponding author on reasonable request.

## Abbreviations

p-AKT: Phosphorylated AKT
DN: Diabetic nephropathy
DM: Diabetes mellitus
ESRD: End-stage renal disease
ALB: Albumin
GBM: Glomerular basement membrane
TGF-β1: Transforming growth factor β1
LPS: Lipopolysaccharide
STZ: Streptozotocin
UUO: Unilateral ureteral obstruction
BUN: Blood urea nitrogen
Scr: Serum creatinine
ELISA: Enzyme linked immunosorbent assay
SD: Sprague Dawley
NC: Normal control
FG: Fasting glucose
INH: LY294002
I.P.: Intraperitoneal
DMSO: Dimethyl sulfoxide
ALT: Alanine aminotransferase
AST: Aspartate aminotransferase
SPF: Specific pathogen free
GLU: Glucose
PAS: Periodic acid-Schiff
IHC: Immunohistochemical analysis
WB: Western blot
PCR: Polymerase chain reaction
mTORC1: Mammalian Target of Rapamycin complex1
ANOVA: One-way analysis of variance
