# Supplementary material for: Celastrol slows the progression of early diabetic nephropathy in rats via the PI3K/AKT pathway
Source: BMC Complement Med Ther. 2020 Oct 23;20:321. doi: 10.1186/s12906-020-03050-y (PMC7583204; doi:10.1186/s12906-020-03050-y)
Supplement: Supplementary file 1 — Additional file 1. [file 12906_2020_3050_MOESM1_ESM.pptx]

## Slide 1
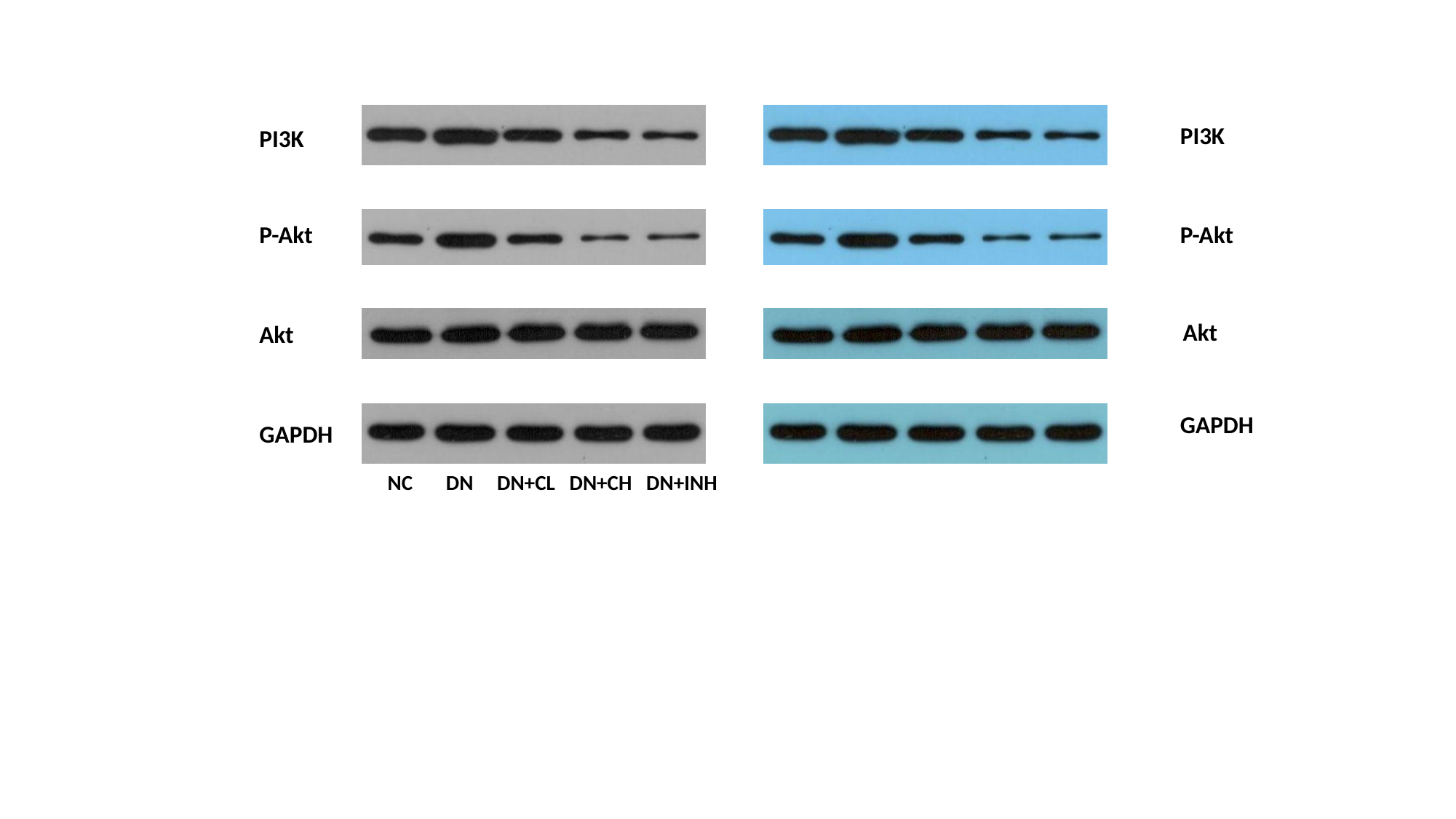

PI3K
PI3K
P-Akt
P-Akt
Akt
Akt
GAPDH
GAPDH
NC DN DN+CL DN+CH DN+INH

## Slide 2
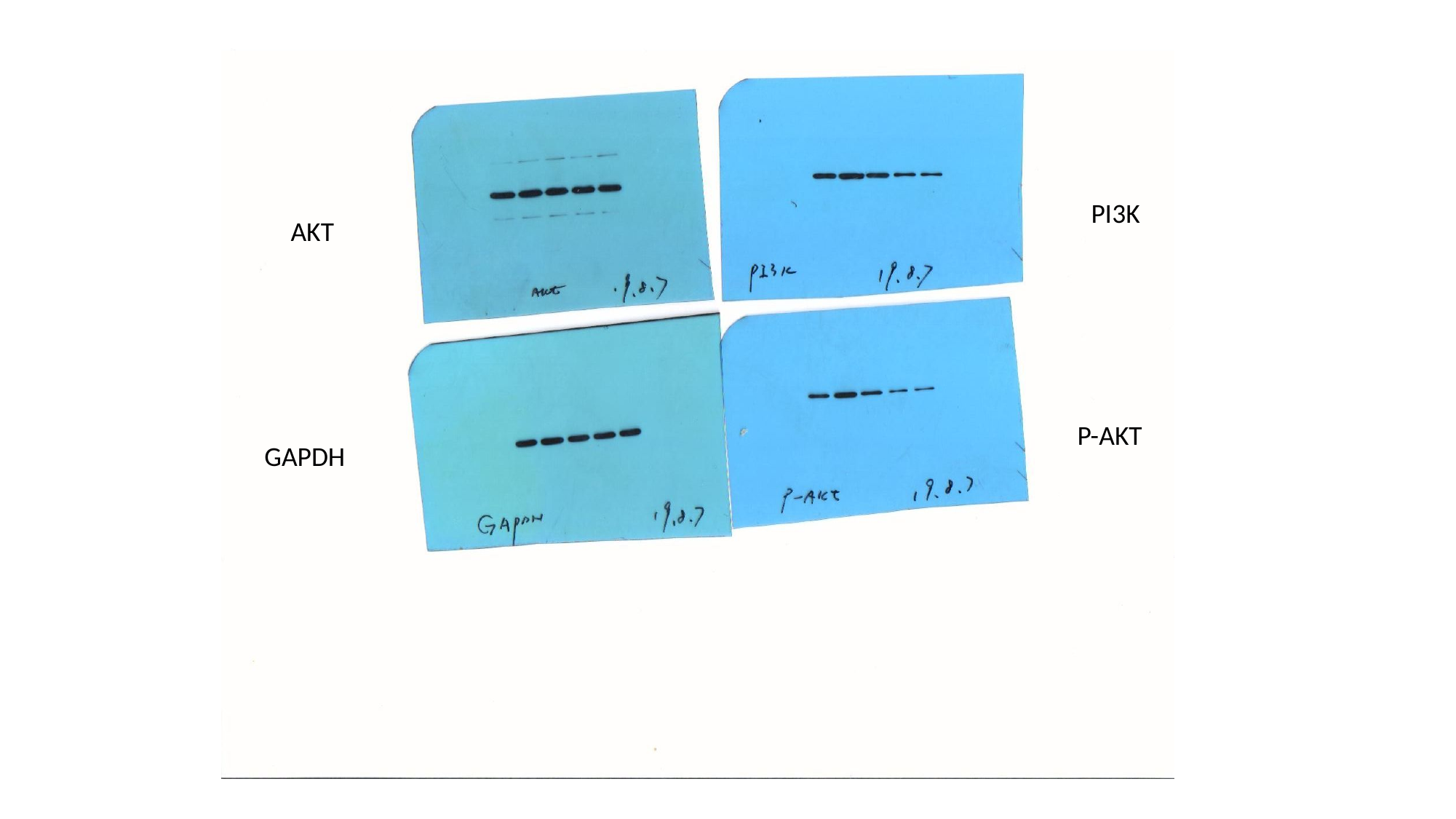

PI3K
AKT
P-AKT
GAPDH

## Slide 3
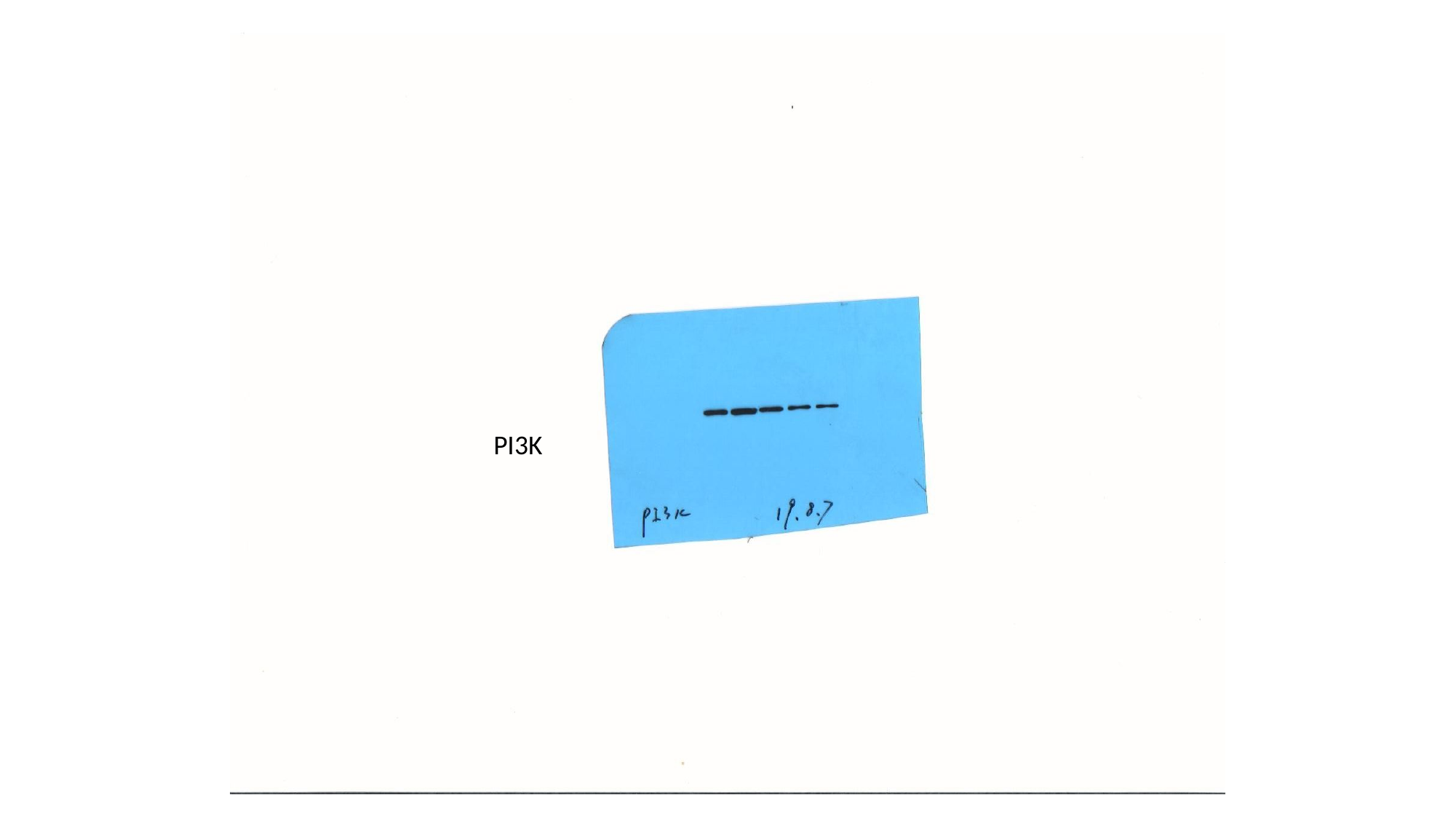

PI3K

## Slide 4
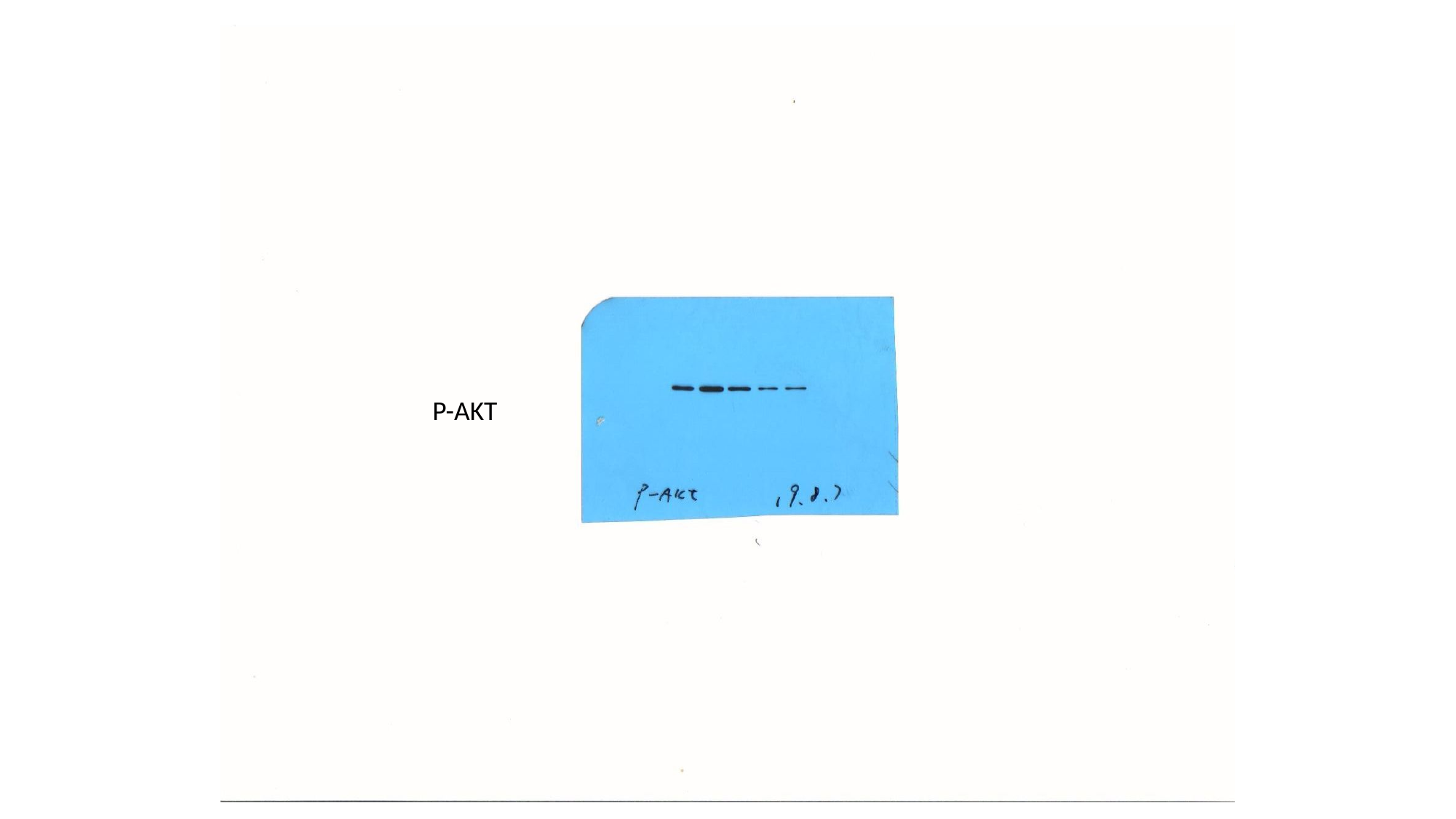

P-AKT

## Slide 5
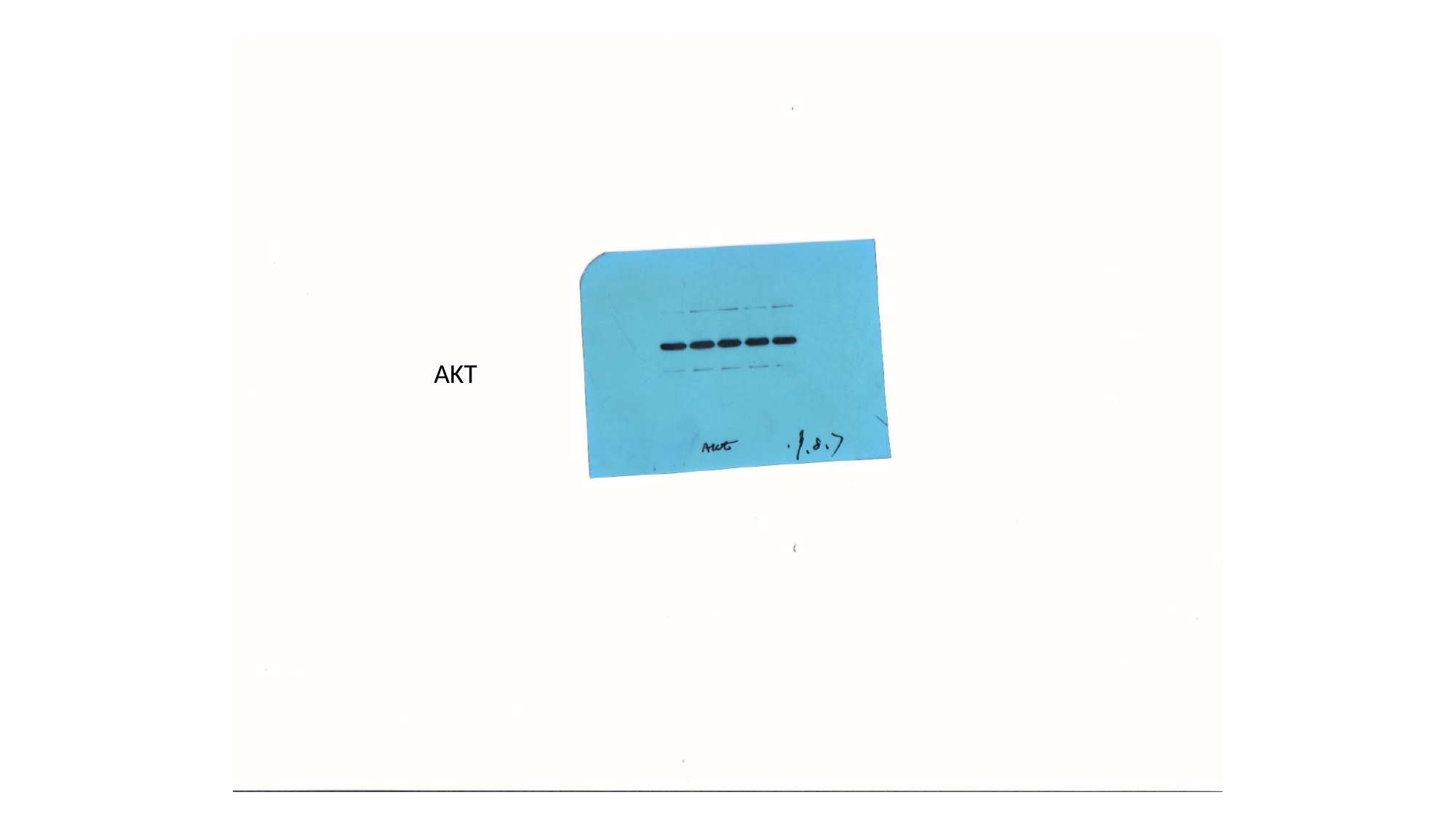

AKT

## Slide 6
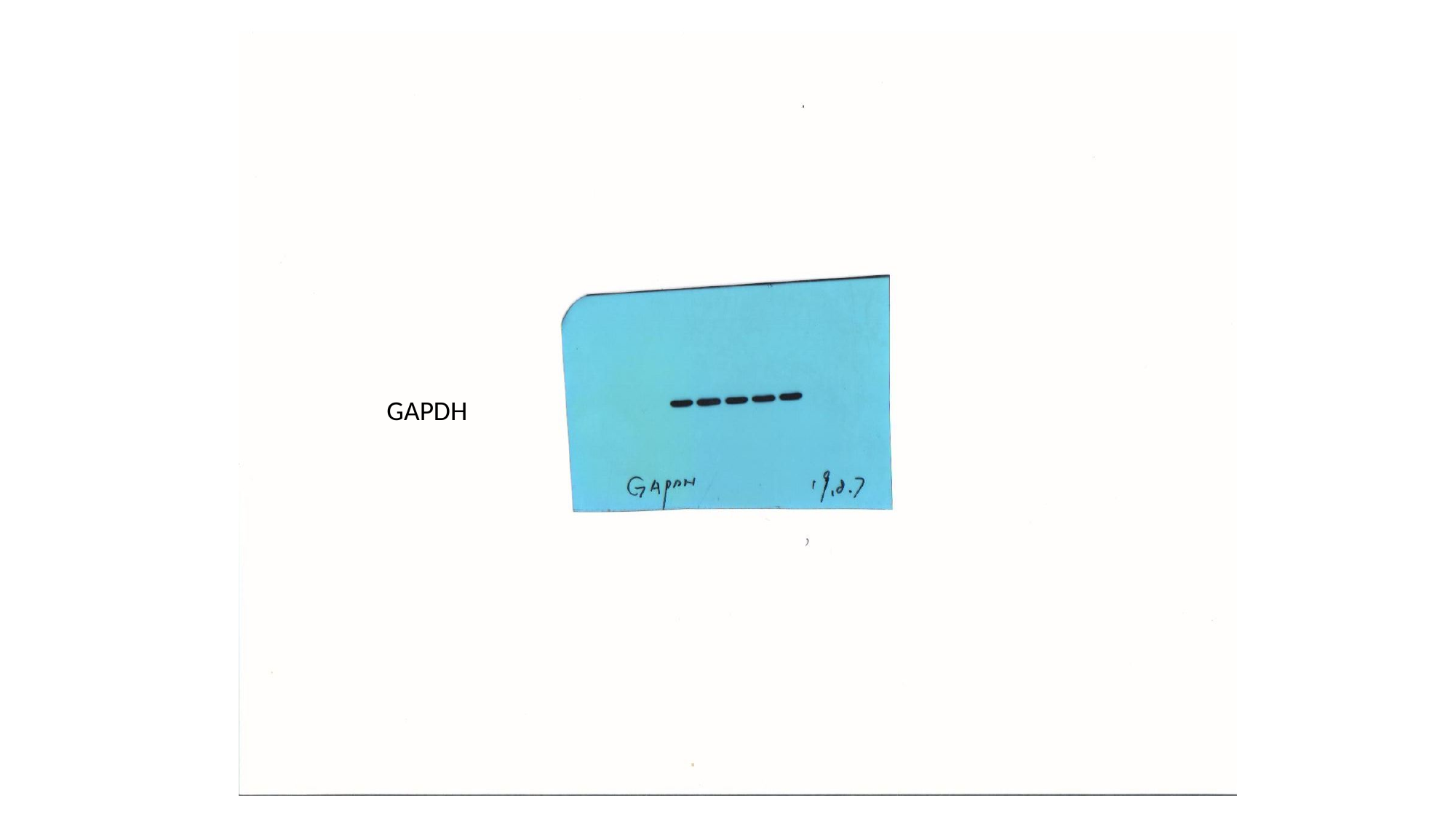

GAPDH

## Slide 7
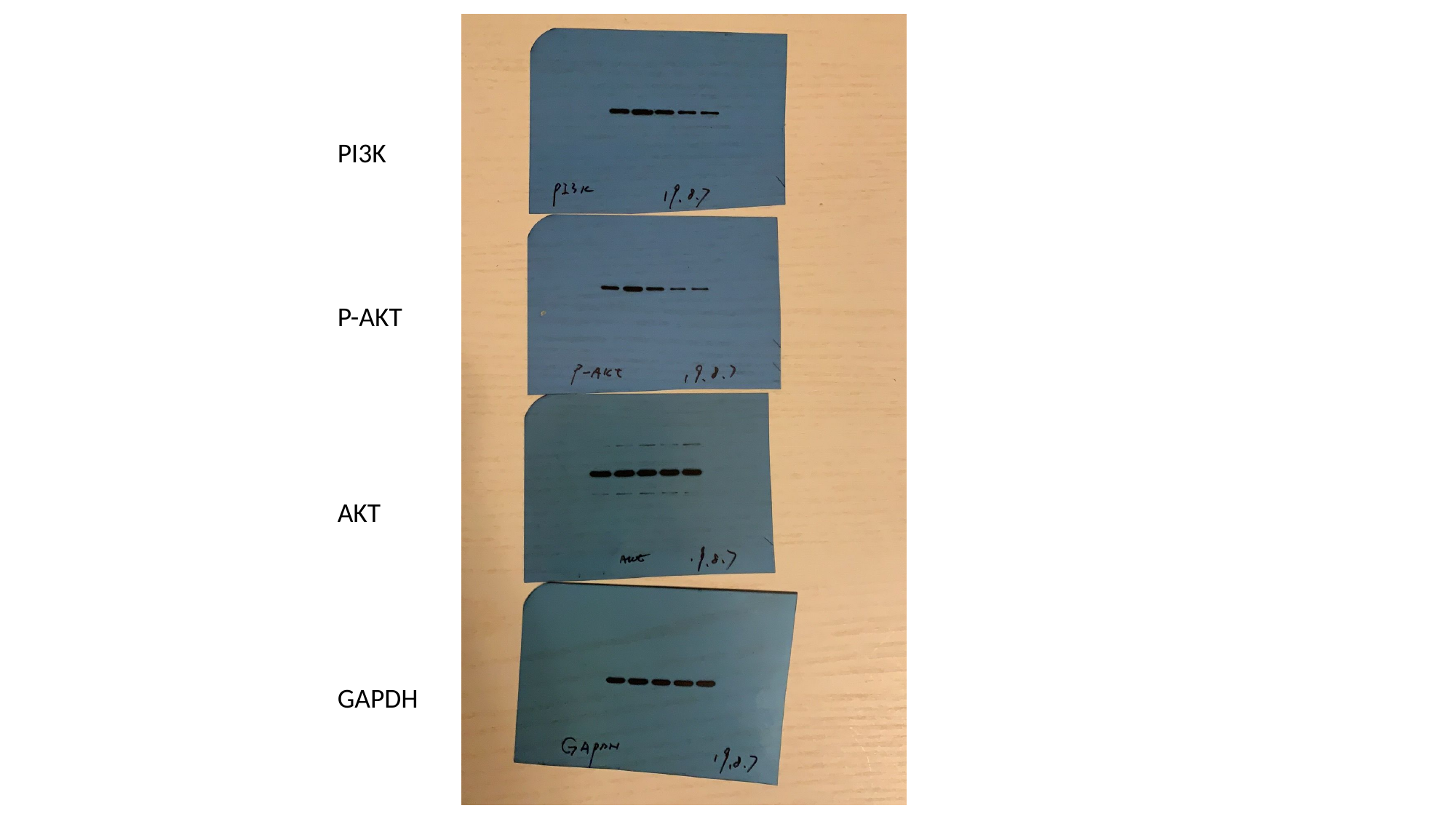

PI3K
P-AKT
AKT
GAPDH

## Slide 8
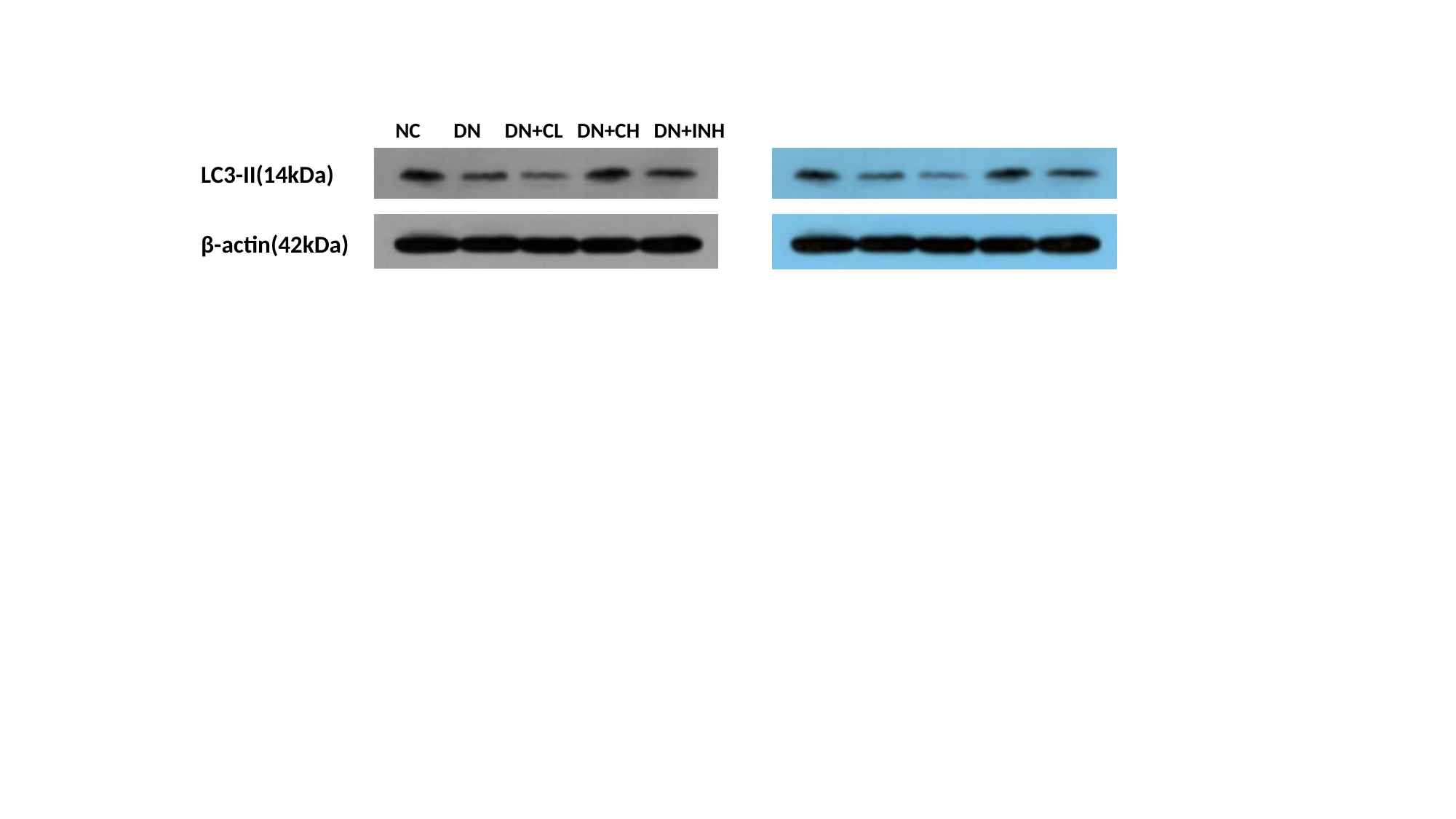

NC DN DN+CL DN+CH DN+INH
LC3-II(14kDa)
β-actin(42kDa)
